# Hypoglycemia with gliclazide MR in women with type 2 diabetes. A continuous glucose monitoring study

**DOI:** 10.1186/1758-5996-7-S1-A61

**Published:** 2015-11-11

**Authors:** André G Daher Vianna, Laiza Tabisz, Claudio Silva de Lacerda, Luciana Muniz Pechmann, Michelle Garcia Polesel, Emerson Cestari Marino, Fellype Barreto

**Affiliations:** 1 Hospital da Cruz Vermelha, Curitiba, Brazil

## Background

Hypoglycemia is usually a limiting factor in the treatment of type 2 diabetes and it may be asymptomatic many times. Sulfonylureas are a group of anti-diabetic drugs associated with an increased risk of hypoglycemia, but, within this group, gliclazide MR seems to be associated with a fewer number of these episodes. Large-scale studies suggest that the incidence of hypoglycemia may be associated with an increase of cardiovascular risk.

## Objective

Evaluate the risk of hypoglycemia by continuous glucose monitoring system (CGMS) in women with type 2 diabetes.

## Materials and methods

A total of 19 post-menopausal women, 60.53±1.08 yrs.-old (mean±SE), with type 2 diabetes for 7.89±1.04 yrs., having poorly controlled diabetes with metformin, were studied. Initial glycated hemoglobin (HbA1c) was 7.28%±0,13%. All patients received additional treatment with gliclazide MR at a dose of 60 or 120mg/day (72.63±5.77mg) for an average period of 6 months. At the end of this period, patients weared a continuous glucose monitoring system (CGMS) for 3.62±0.10 days. The frequency and duration of the hypoglycemia (glucose <70mg/dL) were calculated.

## Results

From a total of 19 women studied, 10 (53%) had at least 1 hypoglycemic episode (mean=0.84 episode/patient/3.6 days using CGMS). From 16 reported hypoglycemic episodes, 94% were asymptomatic. The mean time in the hypoglycemic range among patients studied was 55.53±18.90 min. HbA1c reached at the end of the 6 months was 6.2±0.15%.

## Conclusion

CGMS can provide valuable information for patients and physicians of potentially drug-induced hypoglycemia, especially in sulfonylurea users. In this study, 94% of detected episodes were asymptomatic, which increases their likelihood to cause adverse events. From 3.6 days using CGMS, 55.53 min were within hypoglycemic range. The incorporation of CGMS into the routine of patients with type 2 diabetes, mainly for users of drug that potentially induce hypoglycemia, can result in a more appropriate and safer glycemic control.

